# Prevalence of non-carious cervical lesions and orthodontic treatment: a retrospective study

**DOI:** 10.1186/s40510-022-00409-4

**Published:** 2022-05-16

**Authors:** Rafaella Rodrigues Gomes, Livia Fávaro Zeola, Tiago Augusto Quirino Barbosa, Alfredo Júlio Fernandes Neto, Guilherme de Araujo Almeida, Paulo Vinícius Soares

**Affiliations:** 1 Faculty of Dentistry, Federal University of Uberlândia, Uberlândia, Minas Gerais Brazil; 2 Department of Restorative Dentistry, Faculty of Dentistry, Federal University of Minas Gerais, Belo Horizonte, Minas Gerais Brazil; 3 Department of Occlusion, Fixed Prosthesis and Dental Materials, Faculty of Dentistry, Federal University of Uberlândia, Uberlândia, Minas Gerais Brazil; 4 Department of Pediatric Dentistry and Orthodontics, School of Dentistry, Faculty of Dentistry, Federal University of Uberlândia, Rua Professor Mario Porto, 225, Bairro Lidice, Uberlândia, Minas Gerais 38400-138 Brazil; 5 Department of Dentistry and Dental Materials, Faculty of Dentistry, Federal University of Uberlândia, Uberlândia, Minas Gerais Brazil

**Keywords:** Non-carious cervical lesions, Tooth abrasion, Tooth wear, Tooth movement techniques, Orthodontics

## Abstract

**Background:**

This study aimed to assess the distribution of non-carious cervical lesions (NCCLs) by tooth type, investigate the prevalence of NCCLs in patients undergoing orthodontic treatment, and identify the possible associated factors.

**Material and methods:**

A total of 160 patients were enrolled in this retrospective study. Data on the following variables were collected from pre-and post-orthodontic treatment records: age, sex, Angle’s malocclusion, facial pattern, number of activation sessions, compensatory treatment, and retreatment. Frontal, right and left lateral intraoral photographs of each patient were evaluated to identify the presence or absence of NCCLs in each tooth and assess the distribution of NCCLs in the 3840 teeth from the enrolled patients. Furthermore, patients were classified as NCCLs present, irrespective of the number of NCCLs on the teeth or NCCL absent. Bivariate and multivariate Poisson regression analyses with robust variance were used to assess the association between the NCCLs and each independent variable. Prevalence ratio and 95% confidence intervals were calculated and *p* < 0.05 was considered statistically significant.

**Results:**

The prevalence of NCCLs before and after orthodontic treatment was 22.71% and 30.91%, respectively. Premolars were the most affected teeth, followed by the first molars, canines, and incisors. After statistical analysis, age was found to be the variable factor that influenced the prevalence ratio, with NCCL being the most prevalent when orthodontic treatment was performed in adulthood.

**Conclusions:**

Premolars were most commonly affected by NCCLs. Furthermore, age seemed to contribute to the increased prevalence of NCCLs in adults undergoing orthodontic treatment.

## Background

Non-carious cervical lesions (NCCLs) are characterized by a slow and irreversible loss of mineralized tooth structure at the cemento-enamel junction, unassociated with the presence of microorganisms [1, 2]. NCCLs are standard in modern society, with a prevalence rate of 46.7% among adults worldwide [3]. The etiology is multifactorial, involving an association between friction (tooth wear by attrition or abrasion), corrosion (chemical degradation caused by extrinsic and intrinsic acids), and occlusal stress [4].

Specifically, regarding occlusal stress, different types of load act on the tooth structure; such as occlusal loads, which are static and arise from clenching and swallowing and cyclic loads that occur during mastication [5–7]. Another factor that contributes to undesirable temporary cyclic load is orthodontic treatment [8].

As observed in several three-dimensional finite element studies, the stress concentration caused by the forces from orthodontic treatment force is higher in the cervical region of the tooth than in other areas [9–11]. Therefore, the presence of this stress concentration could advocate the initiation or progression of tooth structure loss in the cervical region through micro-ruptures, thereby making it more permeable and susceptible to other etiological factors [12, 13].

Approximately, 56% of the population has malocclusion and is considered a public health problem that can cause occlusal disorders, compromise the dental esthetics and quality of life [14]. Furthermore, concomitant with the high demand for orthodontic treatment [15] among adults, an increasing number of patients complain of NCCLs, especially in the posterior teeth. However, systematic reviews, meta-analyses, and randomized clinical trials focus on studies that evaluate the efficiency of composites, acids, and adhesives, rather than investigating possible associations between the presence of NCCLs and factors present in modern society, such as orthodontic treatment and advancing age [16, 17].

Therefore, the aims of this study were to: (1) determine the frequency of NCCLs in adult patients who underwent orthodontic treatment according to the tooth type, and (2) evaluate the possible risk factors associated with the prevalence of NCCL. The null hypothesis was that orthodontic treatment does not influence the distribution of NCCLs by tooth type and the possible risk factors have no influence on the prevalence of NCCLs.

## Material and methods

This retrospective study was approved by the Ethics Committee of the Federal University of Uberlândia (#1.382.955). Patient records at a private orthodontic practice in Uberlândia, Brazil, were screened with no influence from the treating practitioner. Various factors were taken into consideration: a population of 271 registered patients at the dental office, confidence interval of 95%, margin of error of 5%, design effect of 1.0, and an anticipated outcome frequency in the population of 50%. Subsequently, 160 patients were included in this study.

According to the eligibility criteria, all records were selected serially and consecutively from 2005 to 2015. A total of 99 women and 61 men were selected, with a mean age of 22.8 years, and 27 activation sessions. The inclusion criteria were: participants who underwent orthodontic treatment with a fixed appliance, presence of permanent dentition from the first molars to first molar at the initiation of the treatment, aged between 10 and 52 years, both sexes, documentation of all the previous orthodontic treatment appointments and digital photographs before and after the treatment. On the contrary, patients who had primary or mixed dentition, periodontal diseases, agenesis of any of the permanent second molars, or without previous treatment documentations, or intraoral, frontal and lateral photographs corresponding to the orthodontic treatment were excluded.

Initially, a data collection form was developed in which each patient was assigned a unique number. Subsequently, patient records were evaluated by a single examiner who obtained the patient demographic information, such as age and sex at the beginning of the comprehensive orthodontic treatment. Furthermore, data related to Angle’s malocclusion, facial pattern, number of activation sessions, compensatory orthodontic treatment performed, and orthodontic retreatment were also collected. In this context, the compensatory orthodontic treatment comprised of any interventions that were aimed to camouflage mild or moderate discrepancies of the maxillomandibular relationship by changing the inclinations of the appropriate teeth.

High-resolution intraoral photographs (3008 × 2000 pixels) up to the first permanent molars of both the arches of the patients, before and after orthodontic treatment were captured and evaluated. All the photographs were taken by the same person, using two Nikon camera bodies (D70 and D7100), a 105 mm AF micro-Nikkor lens, and a half-power two-ring flash (Nikon SB 29 s and Yongnuo YN14EX) over the 10 year treatment period. The frontal photographs were obtained perpendicularly to the buccal surface of the maxillary central incisors, at a focal distance of 0.5 m, a diaphragm aperture of 22, and a velocity of 1/125. The right and left lateral photographs were obtained perpendicular to the buccal surface of their respective upper first premolars under the same parameters, except for the 25° diaphragm opening. Any loss of tooth structure in the cervical region, regardless of the lesion size, was considered as NCCL. Each tooth was classified as having an NCCL present or absent on the basis of the photographs.

For data collection on the presence or absence of NCCLs in the photographs, two examiners were trained and calibrated by a dentist, with 15 years of clinical experience, using a random sample of 16 patients. The Kappa coefficients for the intra-examiner and tooth-to-tooth inter-examiner agreements were 0.91 and 0.8, respectively.

After the calibration phase, photographs of the patients were evaluated by the two examiners to determine the prevalence of NCCLs on each tooth before and after orthodontic treatment. All data obtained were included in the data collection form.

### Statistical analysis

Statistical dataThe collected data were recorded in standardized spreadsheets. Descriptive statistics were used to describe the patient characteristics. Bivariate and multivariate Poisson regression with robust variance was used to determine the association between NCCLs and each independent variable. The level of significance and the confidence interval was set at 5% and 95%, respectively. All analyses were performed using the Stata statistical package (version 14.0; StataCorp LP, College Station, TX, USA).

## Results

**Fig. 1:**
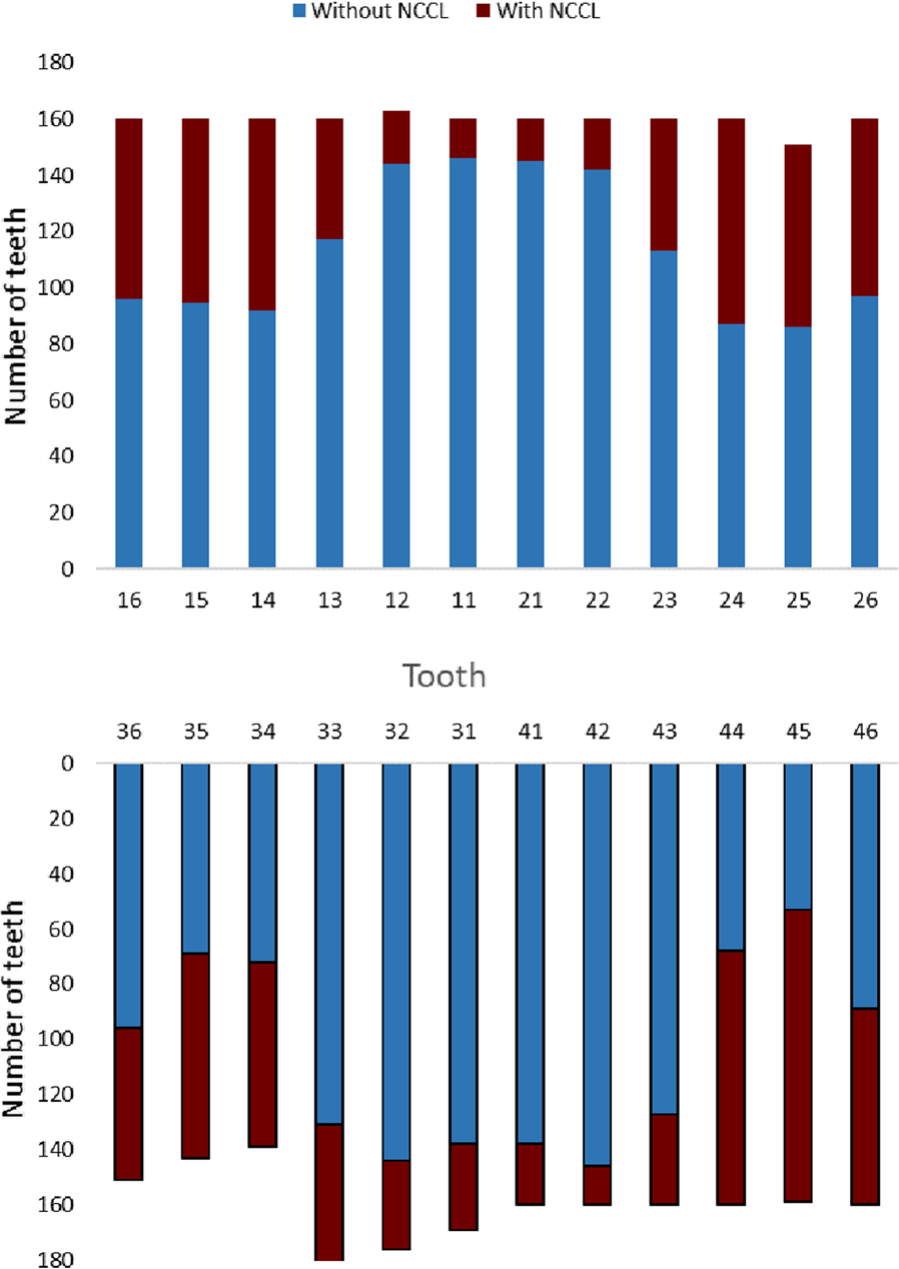
Prevalence of non-carious cervical lesions in permanent teeth subjected to orthodontic treatment (from first molar to first molar)

This study included 160 orthodontic patients and a total of 3840 teeth were evaluated. The prevalence of NCCLs per tooth was 22.71% (872 teeth) before orthodontic treatment and 30.91% (1187 teeth) after treatment, resulting in an increase of 8.2%. The distribution of NCCLs by tooth type is presented in Fig. 1, in which the premolars were the most commonly affected teeth, followed by the first molars, canines, and incisors.

Most of the patients included in this study were women, aged up to 20 years, and presented with an Angle Class I malocclusion and a dolichofacial pattern. The treatment mostly lasted between 19 and 36 sessions, and in most cases, no compensatory treatment or retreatment was performed (Table 1).

**Table 1:** Distribution of variables

Variable	Patients (N = 160)
Gender	
Female	99 (62%)
Male	61 (38%)
Age	
Up to 20 years	94 (59%)
21–35 years	34 (21%)
> 35 years	32 (20%)
Number of activation sessions	
6–18 sessions	27 (17%)
19–36 sessions	113 (71%)
> 36 sessions	20 (13%)
Angle classification	
Class I	76 (48%)
Class II	74 (46%)
Class III	10 (6%)
Facial pattern	
Mesiofacial	54 (34%)
Brachyfacial	10 (6%)
Dolichofacial	96 (60%)
Compensatory treatment	
No	110 (69%)
Yes	50 (31%)
Retreatment	
No	118 (74%)
Yes	42 (26%)

The results obtained from the multivariate-adjusted Poisson regression model are presented in Table 2. This study demonstrated the age promoted changes in the prevalence ratio of NCCLs. Participants aged 21–35 years were 1.14 times (PR = 1.14, 95% CI: 1.05–1.25; *p* < 0.002) more likely to have NCCLs than those aged < 20 years. Moreover, the likelihood of having NCCLs increased 1.19 times for individuals aged > 35 years (PR = 1.19, 95% CI: 1.10–1.29; *p* < 0.001) when compared to younger participants. Contrastingly, sex, Angle’s malocclusion, facial pattern, number of activation sessions, compensatory treatment, and retreatment were not significantly associated with the incidence of NCCLs.

**Table 2:** Poisson regression analyses of the association between non-carious cervical lesions and the independent variables

Variable	PR (95% CI)	*p* value	Adjusted PR (95% CI)	*p* value
Age (reference: up to 20 years)	1		1	
21–35 years	1.13 (1.05–1.23)	0.002*	1.14 (1.05–1.25)	< 0.002*
> 35 years	1.17 (1.08–1.25)	< 0.001*	1.19 (1.10–1.29)	< 0.001*
Gender (reference: women)	1		1	
Men	1.02 (0.94–1.10)	0.564	1.05 (0.97–1.14)	0.158
Angle classification (reference: Class I)	1		1	
Class II	1.05 (0.97–1.13)	0.213	1.00 (0.93–1.09)	0.815
Class III	1.11 (1.00–1.23)	0.046*	1.09 (0.98–1.22)	0.095
Facial pattern (reference: mesofacial)	1		1	
Brachyfacial	1.04 (0.95–1.13)	0.308	1.04 (0.96–1.13)	0.251
Dolichofacial	1.11 (0.98–1.25)	0.079	1.08 (0.95–1.22)	0.198
Number of activation sessions (reference: 6 to 18 sessions)	1			
19–36 sessions	1.03 (0.92–1.15)	0.558	1.07 (0.97–1.18)	0.161
> 36 sessions	1.08 (0.95–1.23)	0.222	1.11 (0.97–1.28)	0.114
Compensatory treatment (reference: no)	1		1	
Yes	0.99 (0.92–1.08)	0.960	0.98 (0.91–1.06)	0.682
Retreatment (reference: no)	1		1	
Yes	1.07 (0.99–1.15)	0.057	1.03 (0.96–1.11)	0.320

*PR* prevalence ratio, *CI* confidence interval

*Significant difference (Poisson regression model, *p* < 0.05)

## Discussion

Considering the study findings, the null hypothesis could not be confirmed. NCCLs increased only 8.2% at the end of the orthodontic treatment. Premolars were the most affected teeth and the age factor influenced the prevalence of NCCLs (Fig. 1).

In recent decades, an increase in the number of adults seeking correction of malocclusion has been reported and is attributed to the growing esthetic demand in the society, comfort of orthodontic appliances, and greater access to information and oral health care by the population [18].

Concurrently, several hypotheses have emerged in the literature to explain the etiology of NCCLs, with a consensus of it being multifactorial and involving the association of friction, corrosion, and occlusal stress factors [4, 5, 12, 19].

One of these hypotheses also states that malocclusion and occlusal trauma/interferences, which possibly increase the stress concentration in the cervical region, contribute to the weakening of the dental structures along with other etiological factors [4, 9–11, 20–22]. The increased stress in this region of the dental crown would vary in intensity depending on the presence of different factors, such as age, type and severity of malocclusion, craniofacial pattern, type of orthodontic movement, treatment duration, root length, amount of bone loss, and orthodontic treatment [18].

In the present study, the prevalence of NCCLs increased from 22.71% to 30.91% after orthodontic treatment. NCCLs were equally distributed in both the upper and lower dental arches, and the most susceptible teeth were the premolars, followed by the first molars, canines, and incisors (Fig. 1). The premolars have lesser crown volume and a considerably thinner buccal bone plate compared to other teeth. Furthermore, they are subjected to excessive non-axial loads during mandibular excursive movements. These factors may lead to higher flexion of teeth and increased stress concentration in the cervical region, thus explaining the increased prevalence of NCCLs (Fig. 1) [22–24]^.^ Furthermore, these findings are consistent with those of previous studies [23, 25–27].

Few variables, such as sex, the type of malocclusion, and especially the craniofacial pattern were expected to influence the prevalence of NCCLs. Different occlusal disorders combined with distinct craniofacial patterns, were expected to generate stress in the cervical vestibular regions of the teeth, and were capable of generating new NCCLs. However, none of these variables were related to the emergence of new NCCLs during orthodontic treatment (Table 2).

In contrast, one of the patient-related characteristics that influenced the prevalence of NCCLs was age. Participants aged 21–35 years and > 35 years were 1.14 and 1.19 times more likely to have NCCLs than those aged < 20, respectively (Table 2). This increase in the prevalence of NCCLs may be due to the extended exposure to etiological factors of NCCLs among older adults and makes them more susceptible to the development of NCCLs during orthodontic treatment [23].

Regarding the characteristics of orthodontic treatment, patients who underwent 6–18 activation sessions showed no statistically significant difference in the prevalence ratio of NCCLs compared to those who underwent 19–36 or > 36 activation sessions (Table 2). According to few studies [28], the number of orthodontic activations depends on the patient compliance, missed appointments and problems/breakage of devices, inadequate oral hygiene, initial malocclusion severity, and age of the individual, thereby significantly affecting the treatment time. As the individual ages, their metabolism tends to slow down. Consequently, the same orthodontic procedure will probably require greater activation and longer time to achieve the desired results, thus prolonging the duration of the treatment [29]. However, despite the longer duration of treatment resulting in extended duration of orthodontic forces acting on the teeth and a more significant number of activations, this variable did not influence the prevalence of NCCLs.

Similarly, individuals who underwent compensatory orthodontic treatment were unlikely to have an increase in the prevalence of NCCLs. However, this result contradicted expectations because changes in the positioning of a group of teeth to compensate for an existing anteroposterior, vertical, and/or transverse skeletal discrepancy modified the axial inclinations of the teeth involved. Changes in the axial inclinations of these teeth could modify the direction of the masticatory forces that act on them, which can generate an increase in the concentration of tension in the cervical region, favoring the development of NCCLs [30]. However, in this study, this finding was not elicited.

In specific situations, such as incorrect diagnoses and planning, poor quality results, muscle imbalance, unfavorable residual craniofacial growth, genetic factors, or inadequate retention protocols, orthodontic retreatment may be necessary [31]. In this study, patients who underwent orthodontic retreatment were no longer susceptible to NCCLs. Although they were subjected to new force applications and respective tooth movements at a slightly older age, these interventions were not sufficient to impact a statistically significant increase in the prevalence of NCCLs.

The lack of statistically significant differences between the prevalence of NCCLs and the number of activations, compensatory treatment, and orthodontic retreatment should not be seen as a total lack of participation of these variables in the development of NCCLs. Their association was investigated only with respect to the ability of each variable in this study to trigger new NCCLs and not their capacity to promote dimensional increases in the pre-existing NCCLs.

This study aimed to analyze patients exclusively by an experienced professional with more than 30 years of experience. The idea of a single professional was adopted to eliminate biases arising from different techniques or levels of training and experience. A limitation of this study was the lack of a control group. Moreover, this was a retrospective study with limited control over the collection of sample variables, which may affect the possible inferences of the association between the etiological factors for NCCL and orthodontic treatment. Therefore, we suggest that further cohort and case–control studies are needed on this topic using more precise methodologies that assess the presence or absence of NCCLs and the possible dimensional changes based on potential etiological factors.

## Conclusion

This study concluded that premolars were most commonly affected by the NCCLs, whereas age seemed to contribute to the increased prevalence of NCCLs in adults undergoing orthodontic treatment.

## Data Availability

The datasets used and/or analyzed in the course of this study are available from the corresponding author on reasonable request.

## Abbreviations

NCCLs: Non-carious cervical lesions
PR: Prevalence ratio
CI: Confidence interval
